# Supplementary figures and images for: Key virulence factors responsible for differences in pathogenicity between clinically proven live-attenuated Japanese encephalitis vaccine SA14-14-2 and its pre-attenuated highly virulent parent SA14
Source: PLoS Pathog. 2025 Jan 7;21(1):e1012844. doi: 10.1371/journal.ppat.1012844 (PMC11741592; doi:10.1371/journal.ppat.1012844)

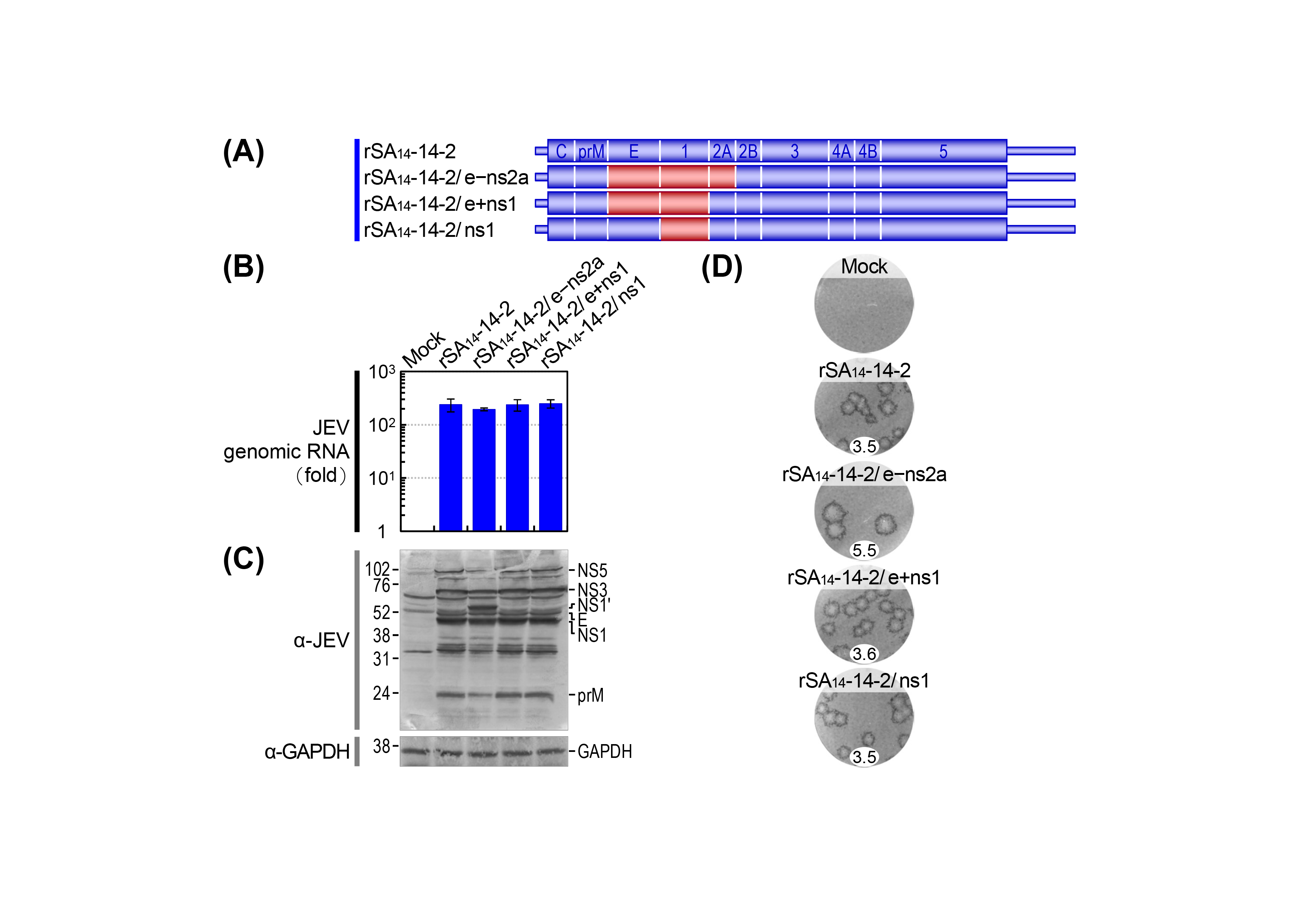

Supplement: S1 Fig — (A) Schematic diagram showing the genomes of rSA14-14-2 and its three derivatives (rSA14-14-2/e–ns2a, rSA14-14-2/e+ns1, and rSA14-14-2/ns1). Colors in the diagram represent the gene(s) of rSA14-14-2 replaced with the corresponding gene(s) of rSA14: rSA14, red; rSA14-14-2, blue. (B-D) BHK-21 cells were either mock-infected or infected with rSA14-14-2 or one of its three derivatives at an MOI of 1. (B) Viral RNA replication. The levels of viral genomic RNA at 20 h post-infection were compared to those at 6 h post-infection by RT-qPCR of total cellular RNAs using a JEV NS3-specific TaqMan probe. The results are presented as fold changes. (C) Viral protein production. The levels of viral proteins at 20 h post-infection were determined by immunoblotting of total cell lysates using a mouse anti-JEV hyperimmune antiserum. GAPDH was used as a loading control. Each blot shows protein molecular weight markers in kDa on the left and viral proteins on the right. (D) Viral plaque morphology. Viral plaques were visualized by immunostaining cell monolayers with a rabbit anti-JEV NS3 antiserum, following a 4-day incubation period under a semi-solid overlay. Representative plaques are shown with their average dimensions (in mm). (TIF) [file ppat.1012844.s001.tif]

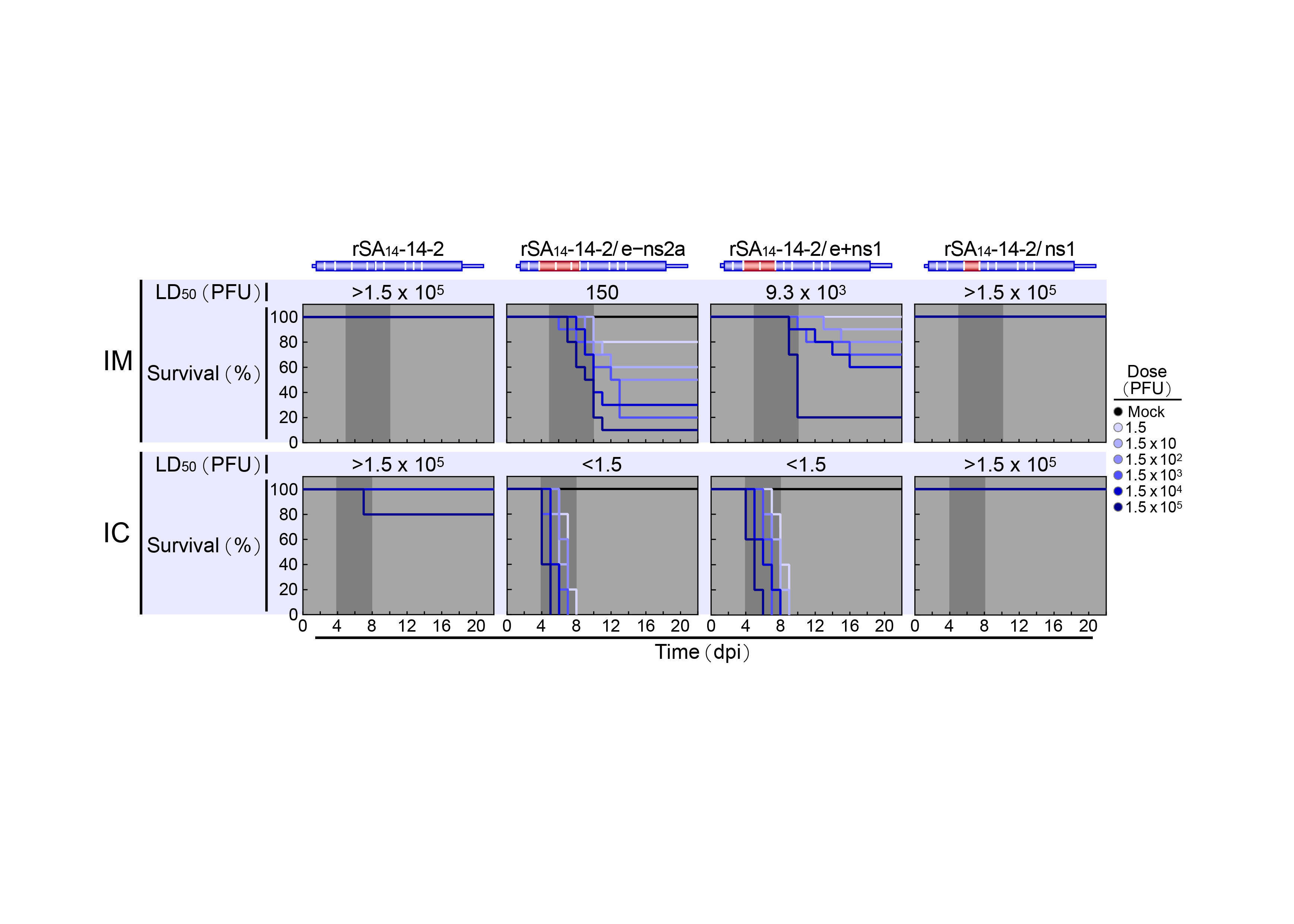

Supplement: S2 Fig — Groups of CD-1 mice were either mock-infected or infected intramuscularly (IM, n = 10 per group) or intracerebrally (IC, n = 5 per group) with rSA14-14-2 or one of its three derivatives detailed in S1 Fig, at a dose ranging from 1.5 to 1.5×10^5^ PFU/mouse. To generate dose-response survival curves, the mice were observed daily for clinical signs and mortality for 22 days after infection. The 50% lethal doses (LD50s) were then calculated from these survival curves. dpi, days post-infection. (TIF) [file ppat.1012844.s002.tif]

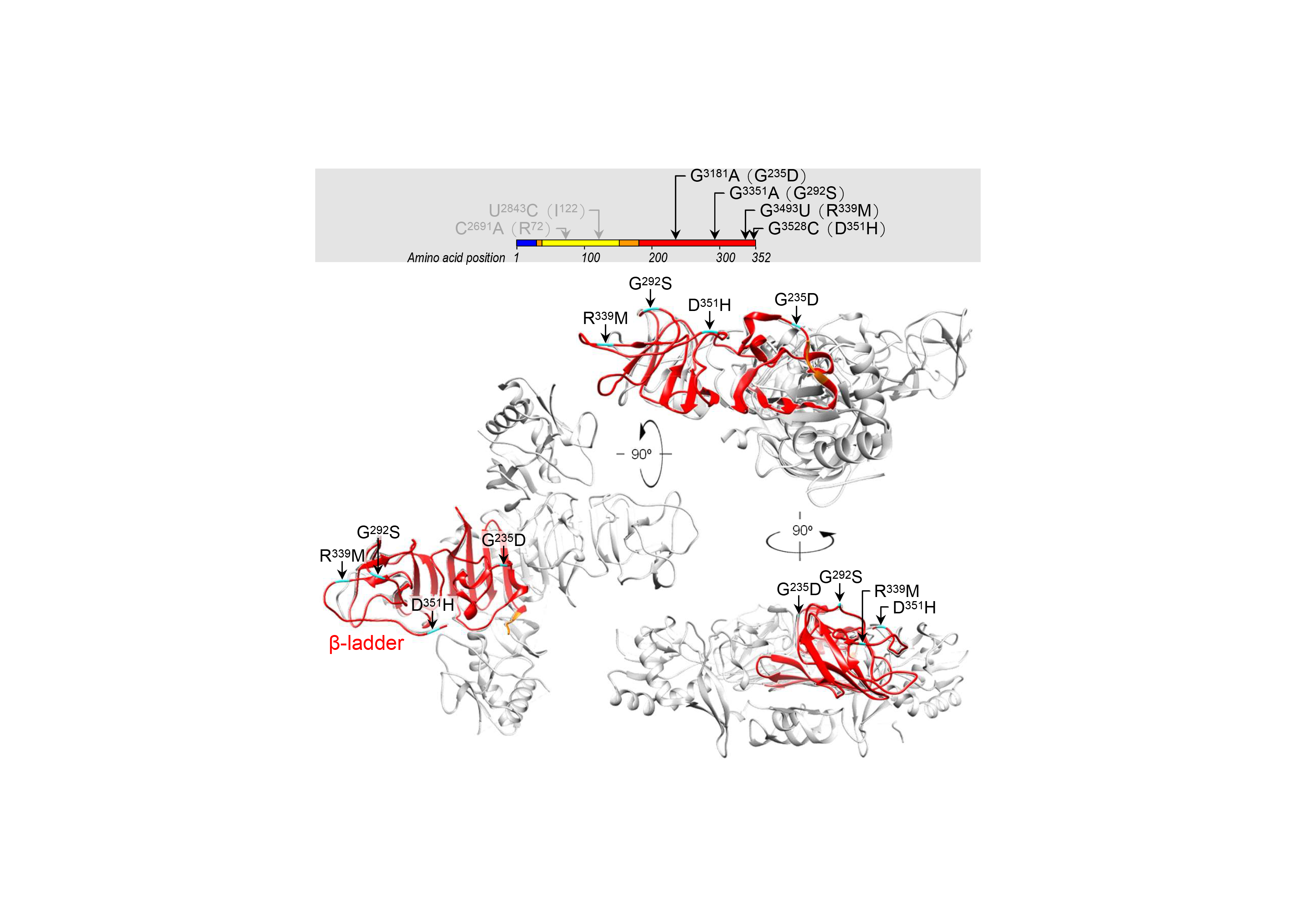

Supplement: S3 Fig — The top panel shows the locations of four missense mutations (in black) and two silent mutations (in gray) found in the primary sequence of the NS1 protein of SA14-14-2, as compared to that of SA14. The nucleotide positions are based on the sequence of the SA14 genomic RNA, and the amino acid positions are based on the sequence of the NS1 protein. The bottom panel maps the positions of the four missense mutations (cyan) onto the crystal structure of the C-terminal β-ladder domain (red, PDB accession code: 5O19) of JEV SA14 [132]. For comparison, the crystal structure of the JEV SA14 β-ladder domain is superimposed on the crystal structure of the full-length NS1 dimer (gray, PDB accession code: 4O6D) of WNV NY99 [126]. (TIF) [file ppat.1012844.s003.tif]
