# Supplementary material for: Key virulence factors responsible for differences in pathogenicity between clinically proven live-attenuated Japanese encephalitis vaccine SA14-14-2 and its pre-attenuated highly virulent parent SA14
Source: PLoS Pathog. 2025 Jan 7;21(1):e1012844. doi: 10.1371/journal.ppat.1012844 (PMC11741592; doi:10.1371/journal.ppat.1012844)
Supplement: Attachment — Submitted filename: Response to Reviewers (R2).pdf [file ppat.1012844.s004.pdf]

Response to Reviewers

[REVIEWER #1'S COMMENTS]

The manuscript submitted by Song et al. (2024) to PLOS Pathogens seeks to elucidate the viral factors that lead to differences in pathogenicity (neuroinvasiveness and neurovirulence) between the attenuated Japanese encephalitis virus (JEV) vaccine strain SA_{14}-14-2 and the virulent parental strain SA_{14}. The authors identified mutational differences between the isogenic strains before utilizing a reverse genetic system to the generate full length infectious cDNAs of said strains (rSA_{14}-14-2 and rSA_{14}). Using the two cDNAs as "genetic backbones", the authors systemically swapped in different genomic regions between the two strains to generate chimeric genomes/viruses. Subsequently, the chimeric viruses were evaluated individually as well as compared to rSA_{14}-14-2 and rSA_{14} in terms of their ability to replicate, produce viral proteins, and alter plaque morphology. The pathogenic capacity of the chimeric viruses was also evaluated in mice. Finally, the authors mapped the identified attenuating mutations onto the structures for envelope (E) protein, non-structural protein 1 (NS1), and non-structural protein 2A (NS2A). Throughout the manuscript, the authors do a thorough job of untangling and identifying the relative contributions of the non-coding regions, E, NS1/NS1' and NS2A proteins on SA_{14} pathogenicity, ultimately providing strong evidence for the E protein serving as a key regulator of neurovirulence and neuroinvasion. This is not a novel conclusion (see Zheng et al. (2018) J Virol, Huang et al. (2024) Virol J, Yu (2010) Vaccine), but is a detailed report on evaluating the contributions including and beyond the E protein. As such, this article would be suited to the pathogenesis and pathogen evolution audience of PLOS Path. Major and minor comments intended to improve the manuscript are outlined in more detail below.

>>> We are grateful for the reviewer's favorable comments. We have rigorously addressed each of the major and minor comments in the revised manuscript, as detailed below. Also, we have included a new reference (Huang et al., 2024, Virol J, 21:128) recently published a few months ago, as mentioned by the reviewer, along with the two papers already discussed in the original manuscript (Zheng et al., 2018, J Virol, 92:e00108-18; Yu, 2010, Vaccine, 28:3635-3641). We hope that this revised manuscript is now improved for the audience of *PLOS Pathogens*, as intended by the reviewer.

[***Reviewer #1, Major Point #1***] Figure 2: It *seems* that the authors have mislabeled the run-off transcription site. Due to the mechanism and positioning of XbaI cleavage, and as the SP6 polymerase uses the reverse ("–") strand as a template to generate the "+" strand, the run-off site is actually tCTAG | not t | CTAG. As such, both genomes contain 4 extra residues (at least based on transcription). Please double-check this, but I think this is correct based on the orientation of your XbaI site as listed. It is unclear to me if you will get deletion of these extra nucleotides upon viral RNA replication since there is a strong bias for the "AG" dinucleotide for initiation of RNA synthesis on both strands in flaviviruses. I don't think this changes the results described herein, but it is good to know that viral RNAs made with extra nucleotides are viable in cell culture.

>>> The reviewer is correct. In the original manuscript, we described in the Materials and Methods section that a full-length JEV cDNA clone was linearized by digestion with *Xba*I and subsequently treated with mung bean nuclease (MBN) to remove the four excess nucleotides of a 5' CTAG overhang left by *Xba*I digestion, as the reviewer noted. We had previously reported this cDNA linearization method to generate infectious run-off RNA transcripts with the authentic 3' end of the viral genomic RNA from a linearized full-length JEV cDNA (Yun et al., 2003, J Virol, 77:6450-6465). However, we accidentally omitted the MBN treatment in the Results section as well as in Figure 2 in the original manuscript. In the revised manuscript, we have added the MBN treatment in the Results section and in Fig 2 and its legend. We now state: "This configuration allows for the *in vitro* run-off transcription of *Xba*I-linearized and mung bean nuclease-treated cDNA ……" [**Results, lines 202-203**] and "Each cDNA clone was linearized by digestion with *Xba*I and subsequently treated with mung bean nuclease (MBN). The linearized cDNA was then used as a template for

in vitro run-off transcription with SP6 RNA polymerase (Pol).” [**Fig 2 legend, lines 1661-1663**].

[***Reviewer #1, Major Point #2***] Figure 4: While presumably the choice is grounded in previous literature, it is not clear in the manuscript why the authors chose to neglect the potential influence on pathogenicity of other segments/genes beyond the NCRs and c-ns2a (adding a statement to this effect would be helpful). According to the sequencing data between the two strains (Figure 1), there seem to be 8 additional missense mutations scattered throughout ns2b, ns3, ns4b and ns5 gene segments. This is not the first study to report this (see reference 112, 114, 117 etc.). It would be helpful for the authors to comment on these in the discussion section.

>>> As suggested by the reviewer, we have added a sentence explaining how the initial two sets of three chimeric viruses each, for a total of six, were constructed: “These reciprocal chimeric viruses were designed to probe the relative contributions of the viral NCRs, the approximately N-terminal half of the viral ORF that includes all structural proteins, and the approximately C-terminal half of the viral ORF that includes most nonstructural proteins, to viral replication in cell culture and pathogenicity in mice.” [**Results, lines 261-264**]. Our data shown in Fig 5 also suggest the potential role of viral genetic factors located in the NS2B-NS5 coding region in viral neuroinvasiveness, which was mentioned in the original manuscript [**Results, lines 303-306 in the revised manuscript**].

>>> Furthermore, as recommended, we have discussed the potential role of the eight missense mutations found in the NS2B-to-NS5 coding region between SA_{14} and SA_{14}-14-2 in the Discussion section: “In addition, there are 29 point mutations, including eight missense mutations (two in NS2B, two in NS3, one in NS4B, and three in NS5), in the NS2B-NS5 coding region between SA_{14} and SA_{14}-14-2. One or more of these mutations have been shown to be involved in viral neuroinvasiveness to some extent in this study, and further investigation is needed to understand their potential roles in viral neuroinvasiveness.” [**Discussion, lines 717-721**].

[***Reviewer #1, Major Point #3***] Discussion, Paragraph 2, “E plays two key roles in JEV pathogenicity: It first acts as the primary initiator for neuroinvasion and subsequently as the sole determinant for neurovirulence”. It would be helpful to have more tie-in/speculation/discussion about how the identified attenuating mutations in E protein may affect entry (and subsequently pathogenicity).

>>> As encouraged by the reviewer, we have discussed how one or more of the nine missense mutations found in the E protein of SA_{14}-14-2 may affect its role in viral entry and pathogenicity. This discussion is now included in the following statement: “Based on our current understanding of the virus [32], its virion structure [35], and viral entry process [37], there are three possibilities for how one or more of the nine missense mutations found in the E protein of SA_{14}-14-2 affect its role in viral entry and pathogenicity: (*1*) They may simply hamper viral entry into a physiologically relevant target cell (e.g., mononuclear phagocytes and neurons). (*2*) They may affect the choice of viral entry pathway, which differentially alters host cell responses to viral infection and subsequently viral pathogenicity. (*3*) They may not affect the choice of viral entry pathway but may alter host cell responses to viral infection, independent of the route of viral entry, subsequently affecting viral pathogenicity. Further research is needed to determine the precise amino acid combinations in the E protein and their underlying mechanisms that influence JEV pathogenicity.” [**Discussion, lines 632-640**].

[***Reviewer #1, Major Point #4***] Line 621/622 seems to be a bit of an overstatement based on the results from this study. While it is true that the NS2A protein appears to play a role in enhancing JEV neuroinvasiveness, the conclusion should read more as it does in the abstract, with NS2A touted to be a “[following E-mediated neuroinvasion, NS2A acts as a] secondary promoter, further amplifying viral neuroinvasiveness”.

>>> As suggested by the reviewer, we have revised our statement. It now reads: “we found that the NS2A protein, along with the NS1/1’ protein, acts as a secondary promoter, amplifying JEV

neuroinvasiveness after E-mediated neuroinvasion." [**Discussion, lines 680-681**].

[***Reviewer #1, Major Point #5***] In the last paragraph, there should be some reference or discussion surrounding the idea that NS2A has been previously associated with increased virulence and disruption of neurogenesis (see Ávila-Pérez et al. (2019) Sci Rep and Yoon et al. (2017) Cell Stem Cell).

>>> As requested by the reviewer, we have discussed the two reports showing that the NS2A protein is associated with viral virulence and the disruption of cortical neurogenesis in mice [**Discussion, lines 699-701**]. The suggested references have been included in the revised manuscript.

[***Reviewer #1, Minor Point #1***] In the Introduction, the authors should consider defining enzymatic domains before jumping to abbreviations: protease/PRO (line 120), methyltransferase/MET (line 126), polymerase/POL (line 127), helicase/HEL (line 128)

>>> We thank the reviewer for picking up on this. In the revised manuscript, we have removed the abbreviations of those enzymatic domains, as they are unnecessary and not mentioned again. We now state: "This proteolytic cleavage is performed by the viral serine protease located in the N-terminal region of NS3, ……" [**Introduction, line 122**] and "The RNA replication is catalyzed by the viral NS5 protein [49], which possesses methyltransferase and guanylyltransferase activity in its N-terminal region and RNA-dependent RNA polymerase activity in its C-terminal region, together with the NS3 protein [50], which has helicase, nucleoside triphosphatase, and RNA triphosphatase activities within its C-terminal region [51, 52]." [**Introduction, lines 128-132**].

[***Reviewer #1, Minor Point #2***] In the Introduction, it would enhance reader understanding of NS1' if there was more explanation provided to speak to its formation (line 122/123). IE "slippery heptanucleotide motif acts as a classic stimulatory motif for -1 ribosomal frameshifting with the downstream pseudoknot hypothesized to reinforce this action and promote a second, short reading frame extending into NS2A. This product is called NS1'. You allude to these components in Figure 1, however without an explanation, the additional labelling may be unclear. Information comes from reference: Firth and Atkins (2009), Virol J.

>>> As suggested by the reviewer, we have added a sentence to explain the formation of the viral protein NS1': "This event is facilitated by the -1 PRF signal, which includes a slippery heptanucleotide motif positioned upstream of a pseudoknot RNA structure [42]." [**Introduction, lines 125-127**].

[***Reviewer #1, Minor Point #3***] Figure 6/7: When stating the results of Figure 6/7, the authors fail to mention the potential impact that NS2A (with N2K and A40V) could be having on plaque size or pathogenicity as there is a no rSA_{14}/NS2A or rSA_{14}-14-2/NS2A. While this question is addressed in Figure 8, there should be some acknowledgement of the lack of this control in the text, but that it will be explored further in the next section.

>>> We appreciate this suggestion. As recommended by the reviewer, we have added a phrase in the revised manuscript: "(see below for a full description of the data on the role of NS2A in viral neuroinvasiveness)" [**Results, lines 390-391**].

[***Reviewer #1, Minor Point #4***] Figure 8/9: The control, rSA_{14}-14-2/e (which was present in the previous figure) is missing from Figure 8/9. Without this control, it makes it difficult to parse apart the contributions of E vs NS2A/prM/C. This is especially evident in Figure 9 whereupon without referring to Figure 6(D), the reader would have little concept of the neuroinvasive synergy that occurs when E and NS2A are replaced together (just the assumption that E alone is causing the changes).

>>> The reviewer's point is well taken. Following the suggestion, we have added a new plaque image of rSA_{14}-14-2/e to Fig 8D, consistent with the image in Fig 6D. The text in the revised manuscript has been updated accordingly [**Results, lines 411-413, 417-420, and 423-426; Fig 8D legend, lines 1758-1760**]. In

addition, we have included a new dose-response survival curve of rSA_{14}-14-2/e in Fig 9, consistent with the survival curve in Fig 7B, IM. The text in the revised manuscript has been modified accordingly [**Results, lines 431, 440-446, and 450; Fig 9 legend, lines 1765-1769**].

[***Reviewer #1, Minor Point #5***] Figure 8: To simplify the results for the reader, the authors should consider re-arranging Figure 8(D). In text, the authors draw the reader to different points of comparison between the plaque assay results - IE "rSA_{14}-14-2/e+ns2a vs. rSA_{14}-14-2/prm+e+ns2a and rSA_{14}-14-2/c+e+ns2a vs. rSA_{14}-14-413 2/c–e+ns2a." Comparisons like this can be difficult to keep track of without visual groupings. It is suggested that either a) the totality of data is shown along with subgroupings of plaque results that the authors would like the reader to compare or b) reorganize the original panel to reflect the comparison groupings that the authors make (IE top down: rSA_{14}-14-2/ns2a, rSA_{14}-14-2/e (see previous comment), rSA_{14}-14-2/ e+ns2a, rSA_{14}-14-2/ prm+e, rSA_{14}-14-2/ prm+e+ns2a, rSA_{14}-14-2/ c+e+ns2a, rSA_{14}-14-2/ c−e+ns2.

>>> As suggested by the reviewer, we have re-arranged Fig 8, panel D according to the second option. As described in our response to Reviewer #1, Minor Point #4 above, we have added a new plaque image of rSA_{14}-14-2/e to Fig 8D, consistent with the image in Fig 6D, to enhance the readability of the main text in the revised manuscript.

[***Reviewer #1, Minor Point #6***] There were several aspects I appreciated about the manuscript and will briefly list a few here: (a) The generation of 20 chimeric viruses was commendable and gave weight to findings in both cell culture and mouse models. (b) It was appreciated that the authors separated out the effects of the viral proteins/NCRs on neuroinvasiveness versus neurovirulence. (c) Figure 1A was easy to digest, clean, and a great reference point for aspects mentioned later in the manuscript.

>>> We are grateful for the reviewer's comments.

[REVIEWER #2'S COMMENTS]

Song et al. describe the construction of infectious clones of virulent parent and live attenuated vaccine strains of JEV. Several sets of chimeras are then created and analyzed for their growth properties in BHK-21 cells and their neuroinvasive and neurovirulent characteristics in CD-1 mice. Genetic determinants influencing these phenotypes are identified in E, NS1 and NS2A. The last section of the Results puts these determinants in the context of what is known about the functions of these proteins and their structures. The quality, scope and rigor of the molecular virology are outstanding, and the paper is very well-written. One could argue that the paper falls short of uncovering the mechanisms by which these determinants influence neurovirulence and neuroinvasion (see below), but the work certainly sets the stage and provides a solid foundation for these kinds of future studies. The current submission represents a great deal of work, but I did find myself wondering about how the JEV parents and the chimeras would behave in an innate immunocompetent cell substrate. However, you could argue that this, again, could be a significant separate study. There is one significant concern regarding neuroinvasion vs neurovirulence. The authors use 2 routes of infection (IM or IC) to interrogate wild-type, vaccine, and all chimeric strains in terms of neurovirulence and neuroinvasiveness. Survival is the only output presented. The results fully support the authors claims for the role of E - NS2A in neurovirulence. However, claims of neuroinvasiveness are neither supported nor unsupported by their data given that the vaccine strain and related derivatives lack lethality after intracranial challenge. It is therefore not possible distinguish between productive peripheral infection followed by non-neuroinvasion vs neuroinvasion followed by non-neurovirulence vs a lack of initial peripheral infection. It is possible that the vaccine strain and derivatives can invade the CNS but are rapidly cleared or the virus is eliminated from the periphery so rapidly that it precludes neuroinvasion.

>>> We appreciate the reviewer's positive and supportive comments. As mentioned, we are very interested in how SA_{14}, SA_{14}-14-2, and their reciprocal chimeric viruses will behave in immunocompromised cells and mice (we believe the reviewer meant "immunocompromised" instead of "immunocompetent"). This is one of our next projects using the SA_{14} – SA_{14}-14-2 system. This project will explore the functional role of innate immunity in the pathogenicity of SA_{14} and the attenuation of SA_{14}-14-2. However, as the reviewer pointed out, this is beyond the scope of the current paper and will be addressed in our next publication.

>>> Regarding the issue of whether SA_{14}, SA_{14}-14-2, and their chimeric mutant viruses can establish a productive peripheral infection prior to neuroinvasion, we have added new experimental data in the revised manuscript, as detailed in our response to Reviewer #2, Major Point below. We hope that these additions address the reviewer's concern.

[*Reviewer #2, Major Point*] To draw conclusions about neuroinvasion, additional assays such as viral titer and genomic RNA levels in the brain and periphery would be needed. Weight curves from the mice would also aid in monitoring disease progression in wild type and vaccine-challenged animals.

>>> As suggested by the reviewer, we have added new experimental data in the revised manuscript. These data show that SA_{14}, SA_{14}-14-2, and their chimeric viruses with varying levels of neuroinvasiveness can clearly establish a productive infection in the spleen at the early stage of viral infection. We now state: "**Demonstration of productive peripheral infections by rSA_{14}, rSA_{14}-14-2, and their chimeric viruses with varying levels of neuroinvasiveness.** A large body of research has established the following infection model [14]: When transmitted by an infected mosquito, JEV first infects mononuclear phagocytes at the bite site of the host's skin. These infected cells, possibly along with cell-free virions, then move to peripheral lymphoid organs, such as the spleen, where JEV replicates in mononuclear leukocytes. This replication causes viremia, leading to a systemic infection that spreads throughout the body, including the CNS. We therefore examined whether a selected set of rSA_{14}, rSA_{14}-14-2, and their chimeric viruses with varying degrees of neuroinvasiveness can cause productive peripheral infections prior to neuroinvasion. To this end, groups of CD-1 mice were infected by IM inoculation with each of rSA_{14} and its derivative rSA_{14}/e, as well as rSA_{14}-14-2 and its six derivatives (rSA_{14}-14-2/e, rSA_{14}-14-2/ns1, rSA_{14}-14-2/ns2a, rSA_{14}-14-2/e+ns1, rSA_{14}-14-2/e+ns2a, and rSA_{14}-14-2/e–ns2a), with rSA_{14} and rSA_{14}-14-2 serving as references. For each virus, we inoculated one-tenth of its IM LD_{50} if it was between 1.5 and 1.5×10^5 PFU (specifically 2.4×10^3 PFU for rSA_{14}-14-2/e, 4.7×10^2 PFU for rSA_{14}-14-2/e+ns1, 28 PFU for rSA_{14}-14-2/e+ns2a, and 15 PFU for rSA_{14}-14-2/e–ns2a), a maximum dose of 1.5×10^5 PFU if the IM LD_{50} was greater than 1.5×10^5 PFU (specifically for rSA_{14}/e, rSA_{14}-14-2, rSA_{14}-14-2/ns1, and rSA_{14}-14-2/ns2a), or a minimum dose of 1.5 PFU if the IM LD_{50} was less than 1.5 PFU (specifically for rSA_{14}). Following the infection, we harvested spleens and brains from four infected mice every two days for 16 days and measured viral loads as PFU per organ (Fig 10). In mice infected with the highly neuroinvasive reference rSA_{14}, we found low levels of viral load (ranging from 20 to 3.1×10^3 PFU/organ) in the spleen from day 2 until day 6, which became undetectable after day 8. In the brain, we found high levels of viral load (ranging from 50 to 1.4×10^7 PFU/organ) from day 6 to day 10, at which point the analysis ended. In mice infected with the non-neuroinvasive rSA_{14}-derived rSA_{14}/e, like its parent rSA_{14}, we also detected 30 to 2.8×10^3 PFU/organ in the spleen from day 2 to day 6, which fell below the detection limit after day 8; however, no virus was detected in the brain during the entire experiment. Importantly, the non-neuroinvasive reference rSA_{14}-14-2 and all its derivatives behaved similarly to rSA_{14}/e, replicating in the spleen yet producing 10 to 8.0×10^2 PFU/organ from day 2 to day 6, which decreased to undetectable levels after day 8. In the brain, however, we did not detect viral replication if their IM LD_{50}s were $>1.5\times10^5$ PFU (for rSA_{14}-14-2, rSA_{14}-14-2/ns1, and rSA_{14}-14-2/ns2a) or we detected it only occasionally, yielding a viral load of 50 to 4.0×10^6 PFU/organ, if their IM LD_{50}s were $<1.5\times10^5$ PFU (for rSA_{14}-14-2/e, rSA_{14}-14-2/e+ns1, rSA_{14}-14-2/e+ns2a, and rSA_{14}-14-2/e–ns2a) from day 4 until the end of the experiment. Taken together, our findings indicate that rSA_{14}, rSA_{14}-14-2, and their chimeric viruses with varying levels of neuroinvasiveness can clearly establish a productive infection in the spleen at the early

stage of viral infection." [**Fig 10; Results, lines 454-485**].

>>> Regarding the body weight changes of the mice, we typically monitored JEV-infected mice for clinical signs, survival rates, and survival times. Based on our experience with JEV SA_{14} and another wild-type JEV strain CNU/LP2 (Kim et al., 2008, J Virol, 82:7846-7862; Yun et al., 2014, PLoS Pathog, 10:e1004290; and Yun et al., 2016, J Gen Virol, 97:2575-2591), JEV-infected mice that show such clinical signs always die within days. This point has been illustrated in the original manuscript: "In all deceased mice, the typical clinical signs of JE, such as lethargy, ruffled fur, hunched posture, tremors, and/or hind limb paralysis, progressively developed over time prior to death, and the virus titers in their brains ranged from 8.4×10^5 to 1.1×10^7 PFU/brain. All of the mock-infected mice (negative control) survived with no signs of disease, as expected." [**Results, lines 243-247**]. While these clinical signs are a good indication of disease progression associated with JEV infection in mice prior to death, body weight changes are not, and therefore we didn't monitor the weight changes in JEV-infected mice in this study.

[*Reviewer #2, Minor Point*] None
